# Innovative tools to advance trachoma elimination in the context of COVID-19

**Published:** 2021-07-20

**Authors:** Scott McPherson, Jude Stern, Simon Bush

**Affiliations:** 1 Scott McPherson Chair, International Coalition for Trachoma Control, RTI International Senior Program Manager for USAID’s Act To End NTDs | East Program, London, UK.; 2 Head of Knowledge Management, International Agency for the Prevention of Blindness, London, UK.; 3 Director of Neglected Tropical Diseases: Sightsavers, Chippenham, London, UK.

**Collaboration in the development and dissemination of new approaches, tools, and resources for mass drug administration is critical during the COVID-19 pandemic.**

**Figure:**
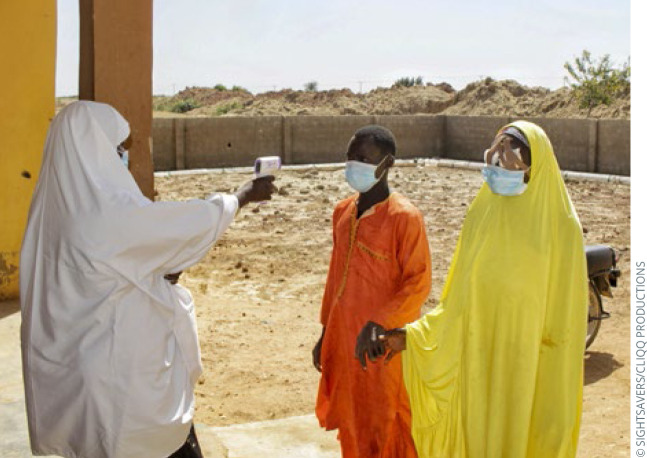
A nurse checks the temperature of a patient and the person accompanying her before they are allowed to enter the hospital. **NIGERIA**

The COVID-19 pandemic has caused significant disruption to the delivery of health programmes around the world. In April 2020, the World Health Organization (WHO) issued interim guidance recommending the postponement of many health-related activities, including community-based interventions that are required to prevent and treat trachoma, the world’s leading infectious cause of blindness. This guidance was updated in July 2020,^1^ recommending programmes only be resumed after thorough risk-benefit analyses and an examination of a list of precautionary measures that should be applied with the aim of limiting the risk of transmission of COVID-19.

Initial analyses by the Neglected Tropical Disease Modelling Consortium projected that, without prompt action and intensified strategies, COVID-19 would disproportionately disrupt disease programmes that use mass drug administration (MDA) as an intervention, including trachoma and onchocerciasis. These neglected tropical diseases (NTDs) affect the world’s most marginalised communities and represent a major challenge to the achievement of universal eye health coverage. The report also projected that trachoma and onchocerciasis would be among the diseases that are more likely to have a resurgence, particularly in areas with the highest disease prevalence, due to the disruption of MDA treatment cycles and other interventions.

To ensure that progress towards universal eye health coverage is not stalled, despite the ongoing pandemic, the International Agency for the Prevention of Blindness (IAPB) launched a COVID-19 taskforce in June 2020 to share experiences, identify challenges, and provide guidance and support to health ministries to mitigate the disruption caused to eye health services. The taskforce identified many shared challenges across eye health delivery, including patient and staff safety, the postponement of interventions such as community-based surveys, active case-finding, and mass treatment campaigns, decreased human resources, disrupted supply chains, travel restrictions, and reduced uptake of interventions due to fear of attending health services. Together, these challenges represent a significant threat to the delivery of eye health services and have the potential to lead to increased vision loss and inequalities.

To assist eye health personnel working with health ministries, health professionals, and programme personnel, the taskforce collated and shared guiding principles, key messages, information, and resources specific to eye health and international development via its COVID-19 and Eye Health Knowledge Hub.^2^ Similarly, in June 2020, the Neglected Tropical Disease NGO Network (NNN) launched a COVID-19 task group to bring implementing partners together to share experiences, lessons learned, and the tools being employed to support health ministries in the safe resumption of NTD programmes.

## Risk assessment and mitigation (RAMA) tools

Health ministries and implementing partners have to make decisions about when and how to safely resume mass drug administration and other NTD-related activities during the COVID-19 pandemic. To support this, a WHO risk assessment and mitigation tool has now been adapted to create a suite of three Excel spreadsheet-based tools for risk assessment and mitigation, collectively known as RAMA. The three tools, developed with funding by United Kingdom Foreign, Commonwealth and Development Office, through the Accelerating the Sustainable Control and Elimination of Neglected Tropical Disease (Ascend) West and Central Africa programme, are available in French and English and cover treatment distribution (including MDA), disease-specific surveys, and – finally – case finding and surgical outreach. There are checklists and questions designed to help regional technical teams and health ministries to identify risk factors, consider how routine NTD activities can be adapted, and review the financial implications of adapting programmes so they are safe during the pandemic.

The RAMA tools have already been used in a variety of programmes across different settings. In the Ascend programme, the tools helped to identify a cost increase of 10–30% associated with programme adaptations such as additional handwashing facilities, active temperature screening, mask wearing, and additional community drug distributors to help with door-to-door drug distribution. They have also helped to identify areas that need improvement. In Ethiopia, RAMA questions helped the federal ministry of health to identify that many communities did not practice proper mask wearing and physical distancing, leading to the recommendation that national health authorities provide further health education to encourage the practice of all COVID-19 precaution measures. In Nigeria, the use of RAMA tools helped programme managers to better understand the benefits of digital platforms, such as WhatsApp and ComCare. These platforms have mitigated the communications challenges posed by restrictions on movement. Notably, community drug distributors took photos to document their adherence to guidelines, such as social distancing, and used these platforms to send the photos to the same supportive supervisors traditionally used during mass drug administration. The digital platforms were embraced by NTD communities in numerous countries to coordinate activities and share lessons learned. Mobile technology also allowed supervisors to access RAMA checklists remotely, which helped to monitor compliance to national COVID-19 guidelines during training and mass drug administration.

## NTD Toolbox

Lessons from the United States Agency for International development (USAID) Act to End NTDs programmes have informed the creation of the new NTD Toolbox (**www.ntdtoolbox.org**). It contains practical approaches to applying WHO guidance to mass drug administration for NTDs in all 26 USAID partner countries across Asia and Africa. The resource provides information to guide communication and coordination for NTD programmes and includes diagrams that illustrate what implementing precautions could look like for different mass drug administration approaches, including distributing drugs house-to-house within a community, during school if the targeted population is school-aged children, and at fixed-point settings within a central gathering point within a community like a town square, mosque or church. It also includes practical approaches for training each type of health care professional within a given national health system on COVID-19 precaution measures and includes ideas and sample checklists for supportive supervision as well as approaches to documenting learning.

**“The development of several vaccines for COVID-19 provides new hope, but it is important that we are not complacent.”**

As national health systems continue to battle the ongoing COVID-19 pandemic, the need to deliver safe eye health services, including those for trachoma, remains critical for ensuring that we do not lose the progress we have made towards elimination. Recognising that eye examinations for trachoma cannot be carried out while maintaining physical distancing, a consortium is currently studying the effectiveness on the reduction of COVID-19 transmission of adding a face shield to the loupes (magnifying lenses) used when assessing trachoma infection and performing trachomatous trichiasis surgery. The work includes assessing the impact of the face shield on the performance of trachoma graders, the comfort and tolerability of the face shield, and the ease and safety of cleaning and re-use.

**Figure:**
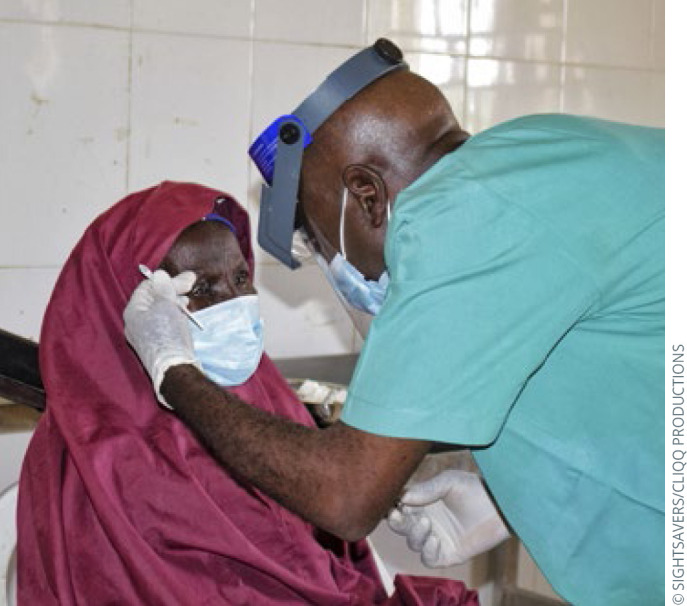
Surgeons wear face shields and face masks at all times, including when removing sutures. **NIGERIA**

WHO’s World Report on Vision and the WHO publication ‘Ending the neglect to achieve the Sustainable Development Goals: A road map for neglected tropical diseases 2021–2030’ emphasise the importance of country-led, integrated, people-centred and cross-sectoral programmes. Through the coordinated efforts of NTD and eye health stakeholders, many programmes have been able to resume safely and effectively without undermining efforts to control the spread of COVID-19.

The development of several vaccines for COVID-19 provides new hope, but it is important that we are not complacent. Continuing the work to eliminate NTDs during an ongoing pandemic will require that eye health and NTD stakeholders, particularly those working in trachoma and onchocerciasis, continue to work collaboratively to develop and disseminate new approaches, tools, and resources that will support health ministries in our new operating context. Such tools are not only critical to respond to COVID-19, but will also strengthen the capacity and resilience of health systems to respond to future health crises through improved coordination between national eye health services and other health services, integrated surveillance systems that monitor eye health issues, training of health professionals to deliver interventions, and sustainable financing to support delivery and access to interventions and services. COVID-19 has demonstrated the importance of strong, resilient health systems and integrated comprehensive approaches to health. Although significant challenges remain to ensure that no one is left behind, the pandemic also presents new opportunities to forge stronger partnerships between trachoma programmes and the eye health community in our pursuit of vision for all and universal health coverage.
